# Synergistic effects of agonists and two-pore-domain potassium channels on secretory responses of human pancreatic duct cells Capan-1

**DOI:** 10.1007/s00424-022-02782-9

**Published:** 2022-12-19

**Authors:** Christiane E. Sørensen, Anna Trauzold, Nynne M. Christensen, Doaa Tawfik, Monika Szczepanowski, Ivana Novak

**Affiliations:** 1 Section for Cell Biology and Physiology, Department of Biology, University of Copenhagen, Copenhagen, Denmark; 2 Section for Clinical Oral Microbiology, Department of Odontology, University of Copenhagen, Copenhagen, Denmark; 3 Institute for Experimental Cancer Research, University of Kiel, Kiel, Germany; 4 Forensic Medicine & Clinical Toxicology Department, Faculty of Medicine, Cairo University, Cairo, Egypt; 5 Department of Pathology Hematopathology Section and Lymph Node Registry, University Hospital Schleswig-Holstein, Kiel, Germany; 6 Department of Medicine II, University Hospital Schleswig-Holstein, Kiel, Germany

**Keywords:** Pancreas, H^+^,K^+^-ATPase, K2P channels, Omeprazole, Riluzole, Histamine

## Abstract

Mechanisms of synergistic agonist stimulation and modulation of the electrochemical driving force for anion secretion are still not fully explored in human pancreatic duct epithelial cells. The first objective of this study was therefore to test whether combined agonist stimulation augments anion transport responses in the Capan-1 monolayer model of human pancreatic duct epithelium. The second objective was to test the influence of H^+^,K^+^-ATPase inhibition on anion transport in Capan-1 monolayers. The third objective was to analyze the expression and function of K^+^ channels in Capan-1, which could support anion secretion and cooperate with H^+^,K^+^-ATPases in pH and potassium homeostasis. The human pancreatic adenocarcinoma cell line Capan-1 was cultured conventionally or as polarized monolayers that were analyzed by Ussing chamber electrophysiological recordings. Single-cell intracellular calcium was assayed with Fura-2. mRNA isolated from Capan-1 was analyzed by use of the nCounter assay or RT-PCR. Protein expression was assessed by immunofluorescence and western blot analyses. Combined stimulation with different physiological agonists enhanced anion transport responses compared to single agonist stimulation. The responsiveness of Capan-1 cells to histamine was also revealed in these experiments. The H^+^,K^+^-ATPase inhibitor omeprazole reduced carbachol- and riluzole-induced anion transport responses. Transcript analyses revealed abundant TASK-2, TWIK-1, TWIK-2, TASK-5, K_Ca3.1_, and KCNQ1 mRNA expression. KCNE1 mRNA and TREK-1, TREK-2, TASK-2, and KCNQ1 protein expression were also shown. This study shows that the Capan-1 model recapitulates key physiological aspects of a bicarbonate-secreting epithelium and constitutes a valuable model for functional studies on human pancreatic duct epithelium.

**Supplementary Information:**

The online version contains supplementary material available at 10.1007/s00424-022-02782-9.

## Introduction

The product of the exocrine pancreas is bicarbonate- and digestive pro-enzyme-rich alkaline fluid, which is essential for the processes of digestion and buffering of acidic chyme that reaches into the duodenum from the stomach. Well-functioning stimulus secretion coupling in pancreatic ducts is a prerequisite for bicarbonate transport, fluid production, and clearance of the secretion from the ductal system. In a physiological setting or in vivo, one expects that different hormonal, neural, and para- and autocrine stimuli cooperate synergistically in the control of epithelial ion movements that determine fluid electrolyte composition and volume according to a person’s prandial state [1, 6]. On the cellular level, coincident receptor stimulation with agonists that either activate intracellular cAMP, or inositol 1,4,5-trisphosphate (IP_3_)/Ca^2+^ signaling, can lead to crosstalk and interactions between these pathways. The resulting synergism enables efficient signal transduction at a fraction of each pathway’s maximal capacity, thereby lowering the risk of overstimulation of downstream effectors [2]. Intracellular cAMP and IP_3_/Ca^2+^ signaling are also the main pathways in the regulation of pancreatic duct secretion. Though synergistic interactions have been explored in exocrine glands, their underlying mechanisms under normal and pathological conditions are less investigated in the human pancreatic duct epithelium. To improve the feasibility of such studies, established human pancreatic duct cell lines could be used as a proxy for pancreatic duct tissue. One example is the human pancreatic duct adenocarcinoma (PDAC) cell line Capan-1, which forms polarized monolayers that have been evaluated as a suitable model for functional studies on human pancreatic duct epithelial cells [69]. Nevertheless, there is currently no detailed description of synergistic agonist responses in Capan-1 monolayers. The first objective of the present study was, therefore, to examine whether Capan-1 monolayers recapitulate the effects of synergistic agonist stimulation and therefore could be used in functional studies on intracellular pathway interactions. The agonists, we tested for this purpose, were chosen for their relevance in pancreatic duct bicarbonate secretion, comprising carbachol, ATP (luminally applied), and histamine as activators of IP_3_/Ca^2+^-mediated signaling. These were applied in combination with either secretin or VIP, which induce cAMP-mediated signaling. The effects of combined versus single stimulation were evaluated by the magnitude of secretory (anion transport) responses in Capan-1 monolayers.

Downstream targets of the above-described signal pathways are multiple ion channels and transporters, which mediate pancreatic bicarbonate secretion in a coordinated way. Although many ion transporters that contribute to the secretion of bicarbonate from pancreatic ducts have been identified, the underlying transport mechanisms are not entirely resolved [46]. Our recent studies have shown that gastric and non-gastric proton pumps (H^+^,K^+^-ATPases) are expressed by human and rodent pancreatic duct cells as well as Capan-1 and other PDAC cell lines [47, 67]. The data indicate that proton pumps play an important role in pancreatic secretion and intracellular pH recovery from experimental acidification [47, 67]. The second objective of the present study was to test whether proton pump inhibition affects the secretory responses of Capan-1 cells stimulated with various agonists. Moreover, we have earlier hypothesized that K^+^ channels could be functionally linked to H^+^,K^+^-ATPases [64]. Such channels may fulfill at least two roles related to proton pump activity; firstly is the transport of K^+^ to the extracellular space, where it is required by H^+^,K^+^-ATPases to secrete H^+^ in exchange for K^+^. Secondly, the K^+^ channel may function as an extracellular pH sensor and together with H^+^,K^+^-ATPases participate in homeostatic pH adjusting mechanisms, particularly under the secretion of the highly alkaline pancreatic fluid. Several K^+^ channel members of the two-pore forming domain family (K2P) of background K^+^ channels display alkaline activation and could thus fulfill such a role. Among these, TASK-2 has been found to be expressed in the exocrine pancreas [12] and the HPAF PDAC cell line [15]. This channel is sensitive both to extracellular and intracellular pH, and it is activated by alkalization, as well as inhibited by acidification [19, 39, 40, 52], which renders it a potential candidate for cooperation with proton pumps and pH-related homeostatic functions. Moreover, we have previously described the functional expression of the pH-sensitive TREK-1 channel in PDAC cells [54], which is a member of the TREK subfamily of K2P channels. TREK-1 is activated by intracellular acidification [34, 51] and could thereby also play a role in pH adjustments that are related to proton export from the intracellular compartment. Thus, the third objective was to use molecular biological techniques, imaging, and electrophysiological techniques, to perform a broad analysis of K^+^ channel expression and function in Capan-1 cells, including but not limited to, K2P channel subtypes. To evaluate further whether different growth conditions may have an impact on K^+^ channel transcript expression, we compared mRNA levels in conventionally cultured Capan-1 cells with those in Capan-1 monolayers. Ussing chamber and voltage dye experiments were used to test the effects of the local anesthetics bupivacaine and lidocaine, non-selective inhibitors of TASK-2, TREK-1, and TREK-2 channels [26, 28, 29, 37, 51, 57], on agonist-stimulated anion transport responses from Capan-1 monolayers. We further explored the effects of the neuroprotective agent riluzole on Capan-1 cell membrane potential and monolayer anion secretion. Riluzole was chosen for these experiments, as it has been previously used to characterize K2P channel activation in a similar model of epithelial ion transport from human airway cells, based on Calu-3 cells [10]. Since pancreatic duct cells are regarded as the origin for the development of pancreatic ductal adenocarcinoma and changes in ion transporter expression are characteristic of pancreatic cancer [55], we discuss our observations of K^+^ channel expression both from a perspective of Capan-1 serving as a model for normal pancreatic duct functions and from the perspective of Capan-1 cells originating from pancreatic cancer. Together, our study demonstrates synergistic effects of combined stimulation with physiologically relevant agonists, including histamine, in anion secretion from Capan-1 monolayers. It further shows that anion transport responses to cholinergic stimulation and riluzole are sensitive to proton pump inhibition in these cells. Lastly, this study demonstrates that Capan-1 cells express multiple K^+^ channels, including members of the K2P family, which we propose fulfill a physiological role in anion secretion as well as potassium and pH homeostasis.

## Methods

### Capan-1 cell culture

Capan-1 cells (ATCC, #HTB-79) were cultured under standard conditions (37 °C and 5% CO_2_) in IMDM (Iscove’s modified Dulbecco’s) media, containing 20% FBS and 1% v/v streptomycin and penicillin. The cells were passaged every 4–7 days. Cells used in the Ussing chamber experiments were grown as epithelial monolayers on collagen-coated polycarbonate membranes in Snapwell Inserts (catalog number 3407, Costar® Snapwell™) as described earlier [69].

### Electrophysiological Ussing chamber measurements on Capan-1 monolayers

Inserts with confluent Capan-1 monolayers on membranes were mounted in a vertical Ussing chamber setup (Model P2300, Physiologic Instruments) with two hemichambers. Electrophysiological measurements were performed as detailed previously [69]. Briefly, the hemichamber with access to the luminal monolayer compartment contained a physiological solution composed of (mM) 120 NaCl, 25 Na gluconate, 1.5 CaCl_2_, 1 MgCl_2_, 10 glucose, 10 HEPES, 1.6 K_2_HPO_4_, and 0.4 KH_2_PO_4_, adjusted to pH 7.4 with NaOH (1 M). The solution was continuously ventilated with air. The second hemichamber with access to the basolateral compartment of the monolayer was filled with sodium bicarbonate containing physiological solution as follows (mM): 120 NaCl, 25 NaHCO_3_, 1.5 CaCl_2_, 1 MgCl_2_, 10 glucose, 10 HEPES, 1.6 K_2_HPO_4_, and 0.4 KH_2_PO_4_, under continuous ventilation with 5% CO_2_ in air to equilibrate pH to 7.4. The chambers were warmed by a heating block to maintain both solutions at 37 °C. The transepithelial potential (*V*_te_) was monitored in the open-circuit mode, intermitted by small current pulses of − 5 μA that induced voltage deflections. The injection current (*I*_inj_) was corrected for the tissue surface area (1.12 cm^2^) and together with the voltage deflections used to calculate transepithelial resistance *R*_te_ (Ω*cm^2^) according to Ohm’s law. *R*_te_ was corrected for background empty chamber resistance by subtraction of a mean (± SEM) value of 58.5 ± 1.96 Ω*cm, measured with 12 chambers from which the cells had been removed. Finally, *V*_te_ and *R*_te_ were used to determine the equivalent short-circuit current per tissue area (equivI_sc_ (μA/cm^2^)); inward currents are given a positive sign. ΔequivI_sc_ (μA/cm^2^) denotes the difference between peak agonist-induced changes of equivI_sc_ and the baseline current. *V*_te_ indicates luminal with respect to the basolateral side, and changes in *V*_te_ from the baseline level are displayed as ΔV_te_ (mV). Ag/AgCl pellet electrodes were used for voltage recordings and Ag wire electrodes for current (*I*_inj_) injection (P2020-S, Physiologic Instruments). Electrodes were filled with 3 M KCl in 4% agar and connected to a pulse generator/signal transducer (AD Elektronik Andreas Bühler, Buchenbach, Germany). All voltage recordings were corrected for electrode offset values. Data were digitized and recorded with the Power Lab/8SP acquisition system by use of Chart 5 software (ADInstruments, Inc.).

### Calcium signals

Capan-1 cells were seeded in Wilco dishes and incubated with 1 μM Fura-2 AM (Invitrogen) for 40 min in a physiological buffer without bicarbonate (-BIC). Following washing and equilibration, cells were placed in a chamber regulated to 37 °C, and images were acquired on a Nikon microscope with 40 × oil objective (NA = 1.30). Fura-2-loaded cells were illuminated at λ_ex_ = 340 nm and λ_ex_ = 380 nm (60-ms exposure time, 1-s intervals) using a TILL Polychrome monochromator. Emission was collected at 510 nm by an image-intensifying, charge-coupled device camera (Andor X3 897, Belfast, UK) and digitized by a FEI image processing system (Thermo Fischer Scientific). The intracellular Ca^2+^ transients were recorded as the ratio of Fura-2 fluorescence signals recorded at 340/380 nm after background corrections. The cells were stimulated with histamine (100 μM) in the presence or absence of diphenhydramine (DPH) (100 μM) and subsequently with the positive control ionomycin (1 μM). We determined the peak response to the agonist, given as the ΔFura-2 (peak increase from the baseline). Only dishes with a positive response to ionomycin were included in the analysis.

### Voltage dye fluorescence measurements of Capan-1 membrane potential (V_m_)

Capan-1 cells, grown under conventional culturing conditions, were loaded with the voltage-sensitive dye VF2.1.Cl [36] (200 nM) similar to earlier described procedures [54, 64]. The VF2.1.Cl dye was a kind gift from R. Tsien. After incubation (20 to 40 min) with the dye in a bicarbonate-free solution (detailed above), *V*_m_ was monitored with a Nikon Eclipse Ti microscope, using a 40 × NA1.4 objective. The fluorophore was illuminated at 2-s intervals for 60 ms at 470 nm excitation wavelength, utilizing a TILL Photonics Polychrome monochromator. Fluorescence emission was collected at 500 to 570 nm using an EMCCD camera (Andor X3 897) and digitized with FEI image processing system (Thermo Fischer Scientific). Membrane voltage (*V*_m_) alterations are presented as fluorescence changes, F/F0 (%), with F0 referring to the average fluorescence value collected during the first minute.

### K^+^ channel mRNA expression analyses

The RNeasy Mini Kit (Qiagen 74104) was used to isolate RNA from Capan-1 cells cultured conventionally on 6-well plates or grown as epithelial monolayers on collagen-coated polycarbonate membranes in Snapwell Inserts. RNA was stored at − 80 °C until use.

### nCounter® ion channel assay (NanoString Technologies®, Seattle, USA)

Nanostring nCounter technology is a sensitive method that investigates gene expression using molecular barcoding of the genes of interest. Each barcode represents an actual mRNA copy in the samples that are directly counted with high accuracy and thus avoiding the amplification bias. nCounter reaction mix comprises a capture tag, a reporter tag, target-specific probes (ion channel genes), and target molecules that hybridize with one another. The reporter tag conveys the signal and the capture tag holds the biotin that interacts in turn with streptavidin. In this experiment, the PDAC-relevant ion channels’ genes (*n* = 101) were investigated in the conventionally cultured and the polarized monolayer phenotypes of the Capan-1 cells. Messenger RNA from the respective cells was isolated using Qiagen RNeasy kit. RNA was set to the concentration of 100 ng of purified total RNA in a 30 μL reaction volume. nCounter analysis used negative controls (*n* = 8), where the mean — in addition to the value of “2” as a standard deviation — was subtracted from the copy number value of all samples. The samples were also normalized to the geometric mean of positive controls (*n* = 6) in addition to 6 different housekeeping genes.

### Reverse transcription PCR for analysis of KCNE1 mRNA expression

A 0.5 µg of RNA isolated from Capan-1 was used in RT-PCR reactions performed with the OneStep RT-PCR Kit (QIAGEN 210212). Primers designed to recognize KCNE1 have been described earlier [60] and were synthesized by TAG Copenhagen A/S (Copenhagen, Denmark) with the following sequences:

Forward 5-GGGCATCATGCTGAGCTACAT-3 and reverse 5-TTTAGCCAGTGGTGGGGTTCA-3. The PCR product was run on an agarose gel and transcript size was compared to a standard marker (Fast Ruler LR Low DNA Ladder, Thermo #SM1103).

### Western blotting analyses

Conventionally cultured Capan-1 cells were lysed in lysis buffer containing (mM) 250 TRIS, 1,25 NaCl, 20 NaF, 50 EDTA, 5% Trizol, and 1 × Sigma fast protease inhibitor (Sigma-Aldrich, Germany). The lysates were centrifuged for 15 min at 15000 g at 4 °C, and the supernatant was stored at − 80 °C until use. Protein concentration was determined in the samples by use of a Bradford assay. Ten to 20 μg of protein was loaded on 10% polyacrylamide precast gels (NuPAGE, Bis–Tris, Invitrogen), separated by electrophoresis, and transferred to a PVDF membrane (Invitrogen). Membranes were blocked in a solution with 3% BSA and 2% skim milk in TRIS-buffered saline containing 0.1% Tween (TBST) (60 to 90 min, room temperature (RT)). The membranes were incubated overnight at 4 °C with primary antibodies against KCNQ1 (1:1000 rabbit polyclonal, APC-022, Alomone Labs), TREK-2 (1:600 rabbit polyclonal, APC-055, Alomone Labs), and TASK-2 (1:2000 rabbit polyclonal, LS-C291082, (LifeSpan BioSciences, Inc.)), followed by rinsing in TBST and treatment with horseradish peroxidase (HRP)-conjugated secondary antibodies (1:2000 to 1:2500 goat anti-rabbit, sc-2004 (Santa Cruz) in TBST with 3% BSA and 2% skim milk). Chemiluminescence of the immunoreactive bands was visualized with EZ-ECL (Biological Industries) on the Fusion FX (Vilber Lourmat) imaging system.

### Immunocytochemistry

Confluent Capan-1 monolayers on membranes were fixed in 4% paraformaldehyde in PBS (phosphate-buffered saline) for 15 min at RT. The preparation was washed and treated with 0.1 M TRIS–glycine to reduce autofluorescence. Cells were permeabilized in PBS containing 0.2% Triton-X-100, rinsed once, and incubated with 5% BSA in PBS to block non-specific binding. Cells were then treated with primary antibodies against TREK-1 (1:50, sc-11557, Santa Cruz), TREK-2 (1:250, APC-055, Alomone Labs), and TASK-2 (1:75, APC-037, Alomone) overnight at 4 °C, followed by incubation with secondary Alexa488 conjugated antibodies (1:200) 1 h at RT. Negative controls were treated identically, except for the omission of primary antibody incubation. In the last steps, F-actin was labeled with Alexa Fluor 647 phalloidin (A22287, Molecular Probes) and nuclei stained with DAPI (4′,6-diamidino-2-phenylindole dihydrochloride, Molecular Probes). Membranes carrying the monolayers were cut out of the inserts and mounted on objective glasses with fluorescence mounting medium (S3023, DAKO). Fluorescence was investigated with a 63 × 1.2 NA objective on a Leica TCS SP5-MP confocal laser scanning microscope (Leica Microsystems, Heidelberg). Leica software was used to analyze and export images as TIFF files.

### Chemicals

Standard chemicals, agonists, and inhibitors were purchased from Sigma-Aldrich (Germany) unless otherwise described. TRAM-34 and the CFTR inhibitor CFTRinh172 were obtained from Sigma-Aldrich. Riluzole, CFTRinh172, and niflumic acid (NA) were dissolved in DMSO. Bupivacaine was dissolved in DMSO or H_2_O. Omeprazole stock solution was prepared in acidified ethanol.

### Statistical analysis

Analyses were performed using GraphPad Prism version 9.4.1 (GraphPad Software, San Diego, USA). Data from monolayer measurements are presented in scatter plots, with each symbol representing an individual monolayer tested. Lines and error bars display means ± SEM (standard error of the mean), and *n* indicates the number of experiments performed. The significance of differences among sample means was tested with unpaired, two-sided *t* tests or Welch’s ANOVA, followed by Dunnett’s T3 post hoc test for multiple comparisons. In one experiment, three outlier values were identified by the use of the two-sided Grubbs’s test (alpha = 0.01) and excluded from the analyses. *P* values < 0.05 were regarded as statistically significant.

## Results

### Physiological agonists induce synergistic responses from Capan-1 monolayers

**Fig. 1:**
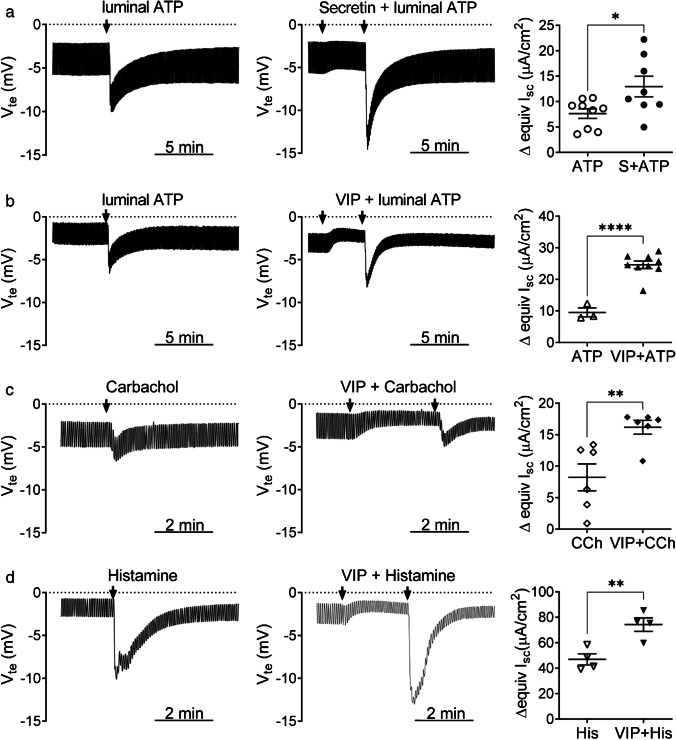
Examples of original *V*_te_ recordings and scatter plots showing peak equivalent *I*_sc_ and *V*_te_ changes in Capan-1 epithelial monolayers, induced by sequential administration of agonists. Prior to single agonist application, monolayers were treated with vehicle instead of the additional stimulant. Apart from ATP, agonists were added to the basolateral compartment. **a** Responses to stimulation of Capan-1 monolayers with secretin and luminal ATP. **b** Luminal ATP induced increase in Capan-1 monolayer responses to VIP. **c** Responses of Capan-1 cells to pretreatment with VIP followed by carbachol stimulation. **d** Effects of VIP pretreatment on histamine responses from Capan-1 cells. Open symbols represent single agonist and filled symbols combined stimulation. Each symbol represents an individual monolayer tested. Lines and error bars indicate mean ± SEM. Number of epithelial monolayers tested for the different conditions: *n* = 3–9. *P < 0.05; **P < 0.01; ****P < 0.0001

First, we investigated whether Capan-1 monolayers reproduce aspects of the complex regulation of native pancreatic duct epithelial cells when subjected to various agonists and whether these cooperate in stimulation of these cells. We therefore tested the hypothesis that hormonal, neurotransmitter, and para- or autocrine agonists act synergistically in the activation of ion transport from polarized Capan-1 monolayers. For this purpose, we monitored the responses to several physiological agents, which act via different intracellular pathways, i.e., cAMP or Ca^2+^ signaling. Transepithelial potential (Δ*V*_te_) changes from baseline towards more lumen-negative values reflect anion secretion and cation absorption across the epithelium. However, no functional epithelial sodium channels (ENaC) have been observed, as tested in earlier studies [44, 69]. In Fig. 1, the left and center panels show examples of *V*_te_ recordings with brief downward deflections evoked by the injection current. Corresponding changes in equivalent short-circuit current (ΔequivI_sc_), which indicate net ion movement across the monolayers, were calculated by Ohm’s law and summarized in the right panel scatter plots. Agonists tested comprised the secretagogue secretin (13 nM), the acetylcholine analog carbachol (10 μM), vasoactive intestinal peptide (VIP) (10 nM), and the para- and autocrine mediators histamine (100 μM) and ATP (10 μM).

**Fig. 2:**
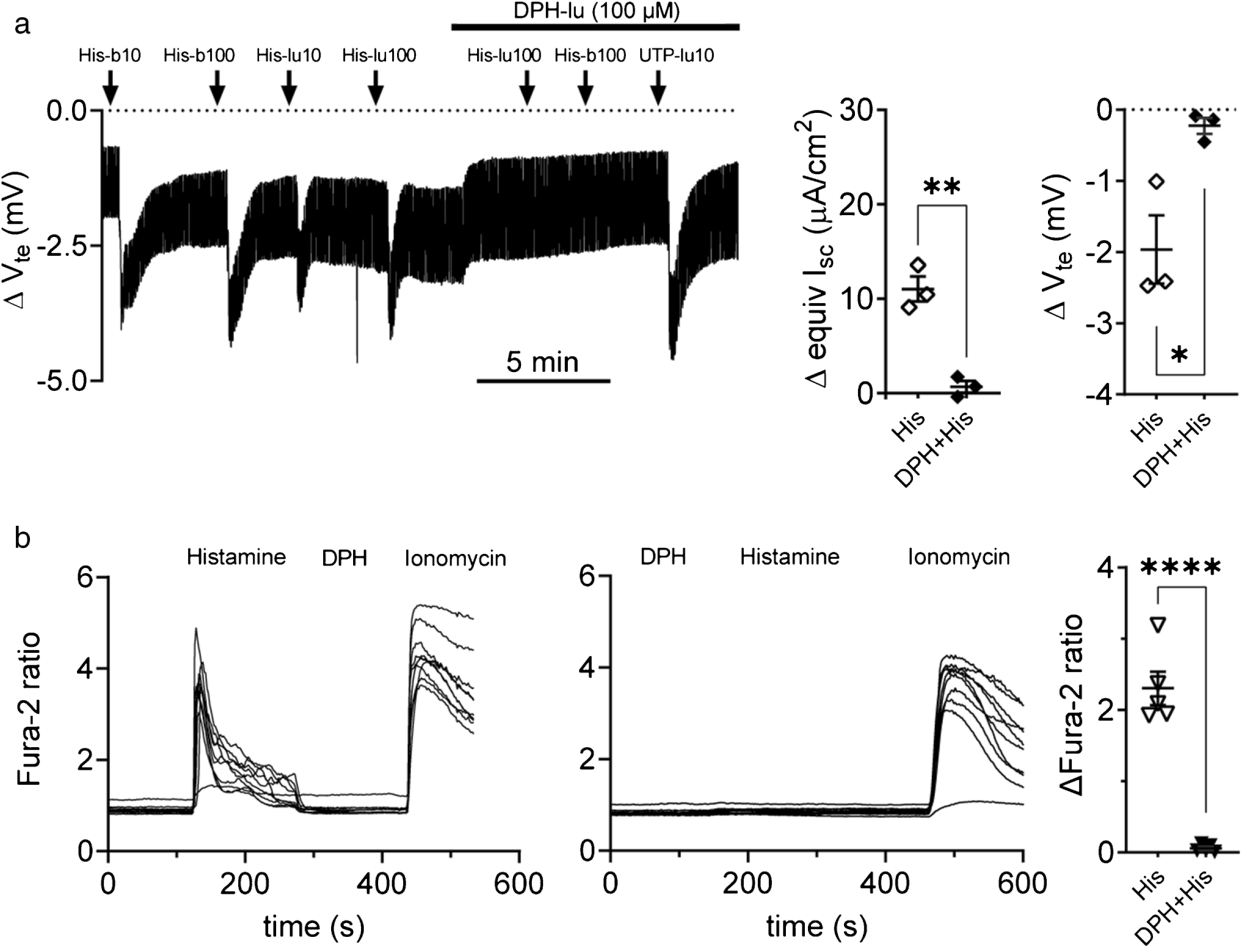
**a** Example of an original *V*_te_ recording and scatter plot of peak equivalent *I*_sc_ and *V*_te_ changes in Capan-1 monolayers (*n* = 3) stimulated with histamine (His, 100 µM) before (open symbols) and after (filled symbols) addition of diphenhydramine (DPH, 100 µM) (lu = luminal, b = basolateral). **b** Example of original Fura-2 ratio fluorescence signals (recorded at 340/380 nm), representing [Ca^2+^]_i_ of single Capan-1 cells (*n* = 10) during histamine stimulation with and without DPH (100 µM) treatment. DPH alone did not induce an increase in Capan-1 [Ca.^2+^]_i_. Scatter plot shows summary of peak Fura-2 ratios from 5 independent experiments, each with 7–10 cells. *P < 0.05; **P < 0.01; ****P < 0.0001

When single agonists were used, the dissolvent of the additional agonists was used as control, before stimulation. Under open-circuit conditions, the mean (± SEM) basal *V*_te_ of all monolayers investigated in Fig. 1a to d was − 1.4 ± 0.1 mV (*n* = 49). The mean basal *R*_te_ and calculated equivI_sc_ of the same monolayers were 530.3 ± 19.7 Ω·cm^2^ and 2.9 ± 0.1 µA/cm^2^, respectively. Combined stimulation of Capan-1 monolayers with secretin and luminal ATP significantly increased the peak ΔequivI_sc_ compared to luminal ATP stimulation alone (Fig. 1a) (13.0 ± 2.0 μA/cm^2^ (n = 8) versus 7.6 ± 0.9 μA/cm^2^, *P* = 0.026 (*n* = 9)). Similarly, pretreatment (2–4 min) of Capan-1 monolayers with VIP augmented the ΔequivI_sc_ response to luminal ATP compared to ATP responses alone (Fig. 1b) (24.6 ± 1.2 (*n* = 9) μA/cm^2^ versus 9.5 ± 1.4 μA/cm^2^ (*n* = 3), *P* < 0.0001). Figure 1c shows that the peak ΔequivI_sc_ induced by carbachol (CCh) was almost twofold increased after pretreatment of Capan-1 monolayers with VIP compared to controls (16.2 ± 1.1 μA/cm^2^ versus 8.2 ± 2.1 μA/cm^2^, *P* = 0.008, (*n* = 6 each)). Finally, Capan-1 monolayers responded to stimulation with histamine from the basolateral compartment by a large increase in *V*_te_, often characterized by a shoulder-formed curve. The corresponding peak change in equivI_sc_ from baseline was also significantly increased after VIP pretreatment of the monolayers (74.3 ± 5.3 μA/cm^2^ versus 46.9 ± 4.4 μA/cm^2^, *P* = 0.007 (*n* = 4 each)). *V*_te_ changes did not vary significantly between pretreated monolayers and controls in the above experiments. Since the effect of histamine is not well explored, we included additional experiments. Figure 2a shows that histamine had large effects on *V*_te_ and equivI_sc_ from the basolateral side, and this effect was abolished with H1 blocker diphenhydramine (DPH). Mean peak ΔequivIsc and Δ*V*_te_ responses to histamine were reduced by 93.8% (*P* = 0.002) and 88.3% (*P* = 0.024 (*n* = 3)), respectively. Figure 2b shows that histamine evokes calcium transients in Capan-1 cells, and these are inhibited by pretreatment with the blocker. Together, these data indicate that Capan-1 cells express functional H1 receptors that couple to the secretory machinery.

### Effects of omeprazole, K2P channel inhibitors, and alkaline pH on Capan-1 agonist responses

**Fig. 3:**
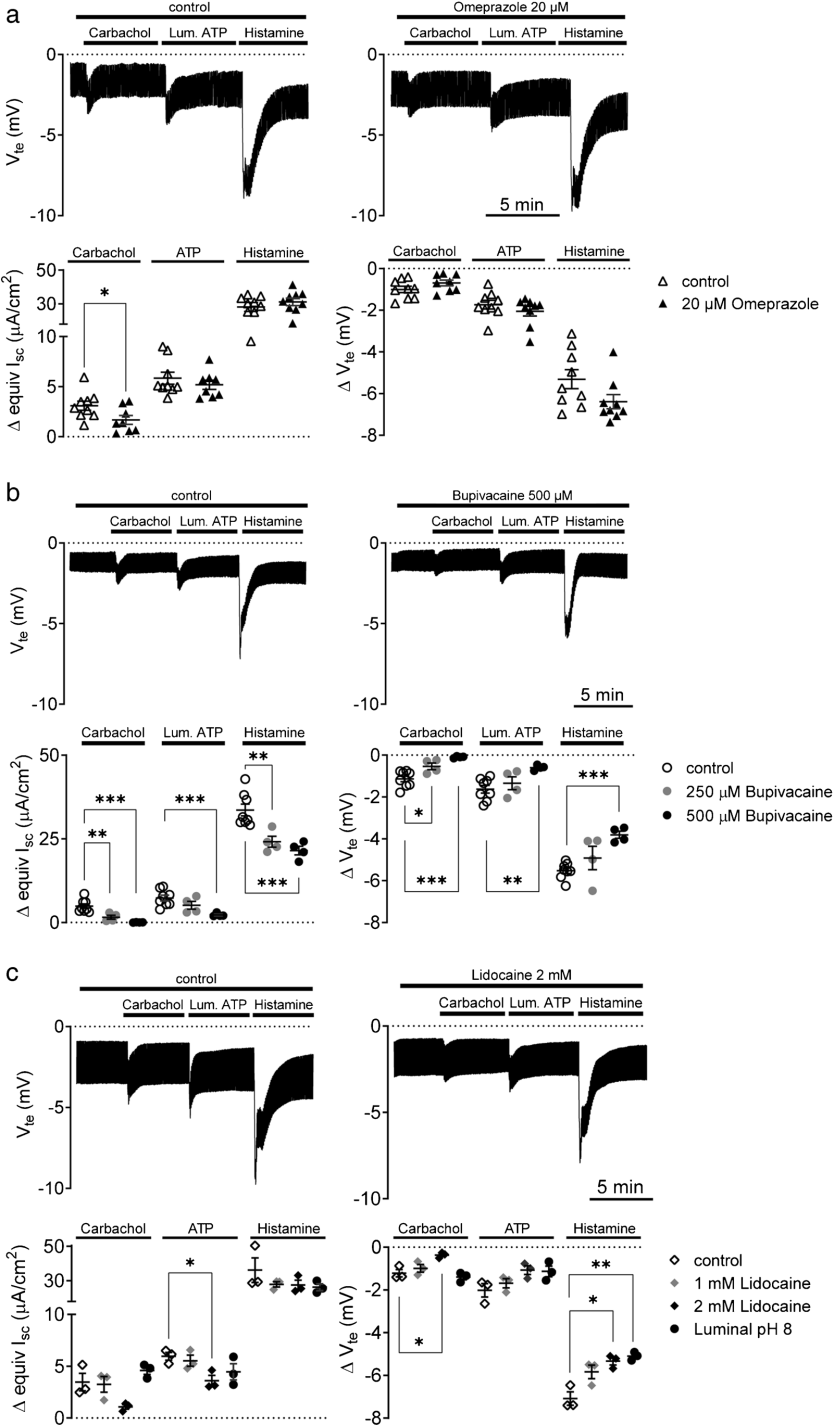
**a** Effects of omeprazole pretreatment on agonist-stimulated luminal anion transport. **b** Effects of bupivacaine (250 or 500 µM) and **c** lidocaine (1 or 2 mM) and alkaline luminal pH (pH = 8) on Capan-1 monolayer responses to physiological agonists. Number of epithelial monolayers tested for the different conditions: *n* = 3–9. *P < 0.05; **P < 0.01; ***P < 0.001

The next experiments were performed to investigate the functional role of H^+^,K^+^-ATPases in anion secretion of Capan-1 monolayers. To examine whether agonist responses of Capan-1 monolayers are affected by inhibition of the gastric H^+^,K^+^-ATPase, we tested the effects of luminal pretreatment with the proton pump inhibitor omeprazole (20 μM) (Fig. 3a) on equivI_sc_ and *V*_te_ changes. In these experiments, the cells were stimulated by sequential administration of carbachol (10 μM), luminal ATP (10 μM), and histamine (100 μM), which in the first series of experiments induced the most consistent electrogenic responses within physiologically relevant concentrations (Fig. 1). Since electroneutral transport of H^+^,K^+^-ATPases cannot be measured in the Ussing chamber setup, effects of omeprazole were evaluated as changes in agonist responses under influence of the inhibitor. Pretreatment with luminal omeprazole (20 µM) significantly reduced the peak carbachol-induced equivI_sc_ change compared with the control (1.7 ± 0.4 μA/cm^2^ (*n* = 8) versus 3.1 ± 0.5 μA/cm^2^ (*n* = 9), *P* = 0.043) (Fig. 3a). In contrast, the equivI_sc_ responses to ATP and histamine, as well as *V*_te_ changes induced by the agonists, showed no significant alterations.

Next, we wanted to establish whether inhibition of K2P channels could be functionally relevant in stimulated anion secretion from Capan-1 monolayers and indicate a pH sensor that may cooperate with H^+^,K^+^-ATPases. Figure 3b and c shows data on the changes in agonist responses after pretreatment with two amide local anesthetics, bupivacaine, and lidocaine, respectively. Pretreatment with luminal bupivacaine (250 or 500 µM) affected electrogenic responses to all three agonists, compared with untreated controls (Fig. 3b (*P* = 0.001 (Welch’s ANOVA test)). With respect to carbachol stimulation, ΔequivI_sc_ changed from 4.9 ± 0.7 μA/cm^2^ (*n* = 8) (control) to 1.6 ± 0.6 μA/cm^2^ (*n* = 4) (*P* = 0.009) after treatment with 250 µM bupivacaine and to 0.03 ± 0.03 μA/cm^2^ (*n* = 4) (*P* = 0.0003) after treatment with 500 µM bupivacaine. ΔV_te_ was after pretreatment with 250 µM bupivacaine reduced from − 1.1 ± 0.1 mV (*n* = 8) (control) to − 0.5 ± 0.2 mV (*n* = 4)(*P* = 0.048) and to − 0.1 ± 0.02 mV (*n* = 4) (*P* = 0.0003) with 500 µM bupivacaine. The luminal ATP-stimulated equivI_sc_ and *V*_te_ changes were significantly reduced only by pretreatment with 500 µM bupivacaine. Thus, the ΔequivI_sc_ changed from 7.3 ± 0.9 μA/cm^2^ (*n* = 8) to 2.3 ± 0.3 μA/cm^2^ (*n* = 4) (*P* = 0.001), and *V*_te_ declined from − 1.6 ± 0.2 mV (*n* = 8) to − 0.6 ± 0.1 mV (*n* = 4) (*P* = 0.002). Histamine-stimulated equivI_sc_ changes were significantly reduced by both concentrations of bupivacaine. ΔequivI_sc_ was reduced from 33.6 ± 1.8 μA/cm^2^ (*n* = 8) to 24.1 ± 1.6 μA/cm^2^ (*n* = 4) (*P* = 0.0065) with 250 µM and to 21.5 ± 1.3 μA/cm^2^ (*n* = 4) (*P* = 0.001) with 500 µM bupivacaine, respectively. The *V*_te_ response was significantly lower after treatment with 500 µM bupivacaine and changed from − 5.5 ± 0.1 mV (*n* = 8) (control) to − 3.8 ± 0.2 mV (*n* = 4) (*P* = 0.0002). The magnitude of inhibition by bupivacaine seemed to be concentration-dependent since pretreatment with half of the concentration affected equivI_sc_ and *V*_te_ peak responses less efficiently.

Lidocaine application to the monolayers led to a significant reduction in the basal current (*P* = 0.031) prior to the stimulation protocol. The inhibitor also appeared to lower Capan-1 monolayer responses to the three agonists (Fig. 3c). However, the Welch’s ANOVA test showed a tendency (*P* = 0.063) only for the effect of 2 mM lidocaine on the ATP-stimulated peak ΔequivI_sc_, which, according to the multiple comparison test, declined significantly from 6.0 ± 0.4 μA/cm^2^ (*n* = 3) to 3.6 ± 0.5 μA/cm^2^ (*n* = 3) (*P* = 0.037). Lidocaine reduced the *V*_te_ responses to carbachol and histamine (*P* = 0.033 and *P* = 0.032, respectively (Welch’s ANOVA test)). In the concentration of 2 mM, the inhibitor changed carbachol-induced *V*_te_ responses from − 1.2 ± 0.2 mV (*n* = 3) to − 0.4 ± 0.1 mV (*n* = 3) (*P* = 0.036) and histamine-induced *V*_te_ responses from − 7.1 ± 0.3 mV to − 5.3 ± 0.2 mV (*n* = 3) (*P* = 0.029). Moreover, in the same experimental series, we tested whether alkalinization of the luminal solution towards pH 8 alters Capan-1 monolayer responses to the above agonists. Compared to the same control as in the experiments with lidocaine, luminal alkalinization reduced the histamine-stimulated *V*_te_ response significantly (from − 7.1 ± 0.3 mV to − 5.1 ± 0.1 mV (*n* = 3), *P* = 0.004 (*t* test)).

### Capan-1 responses to the neuroprotective agent riluzole studied with a voltage dye

**Fig. 4:**
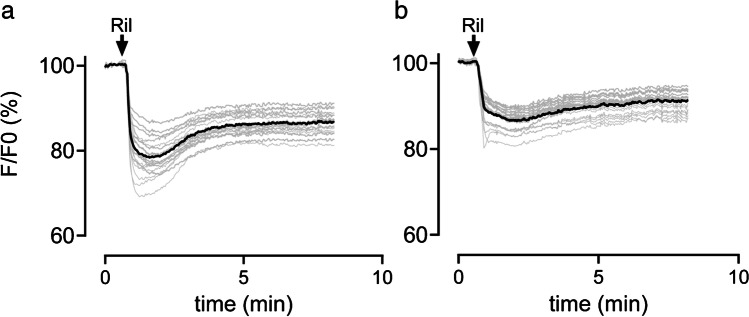
Fluorescence intensity changes in Capan-1 cells loaded with the voltage dye VF2.1.Cl. Cells were stimulated with riluzole (Ril, 100 µM) without (**a**) or with (**b**) preincubation (5 or 15 min) with bupivacaine (500 µM). Light gray lines represent fluorescence measured from single cells. The bold line shows the average fluorescence intensity of all measured cells. Number of experiments for each condition: *n* = 2

To examine whether the inhibitory effects of the two local anesthetics in the previous experiments (Fig. 3b and c) involve K2P channel inactivation, we used a pharmacological agonist to induce K^+^ channel activation in Capan-1 cells and test its sensitivity to bupivacaine. Thus, in the next series of experiments, we investigated whether Capan-1 cells respond to the neuroprotective agent riluzole, which has been previously used to study K2P channel activity in a human airway epithelial model [10]. Capan-1 cells, loaded with voltage dye, were stimulated by the addition of riluzole (100 μM) to the extracellular solution, which induced fluorescence changes (Fig. 4a and b) consistent with a hyperpolarization of *V*_m_ that was maximal approximately after 30 s and followed by slight depolarization. Preincubation of the cells with bupivacaine (500 μM) for 5 or 15 min (Fig. 4b) reduced the mean maximal hyperpolarization (Fig. 4, bold line) by 37.7%. In both cases, *V*_m_ did not return to prestimulation levels under the present experimental conditions (standing bath).

### Effect of inhibitors on responses of Capan-1 monolayers to riluzole

**Fig. 5:**
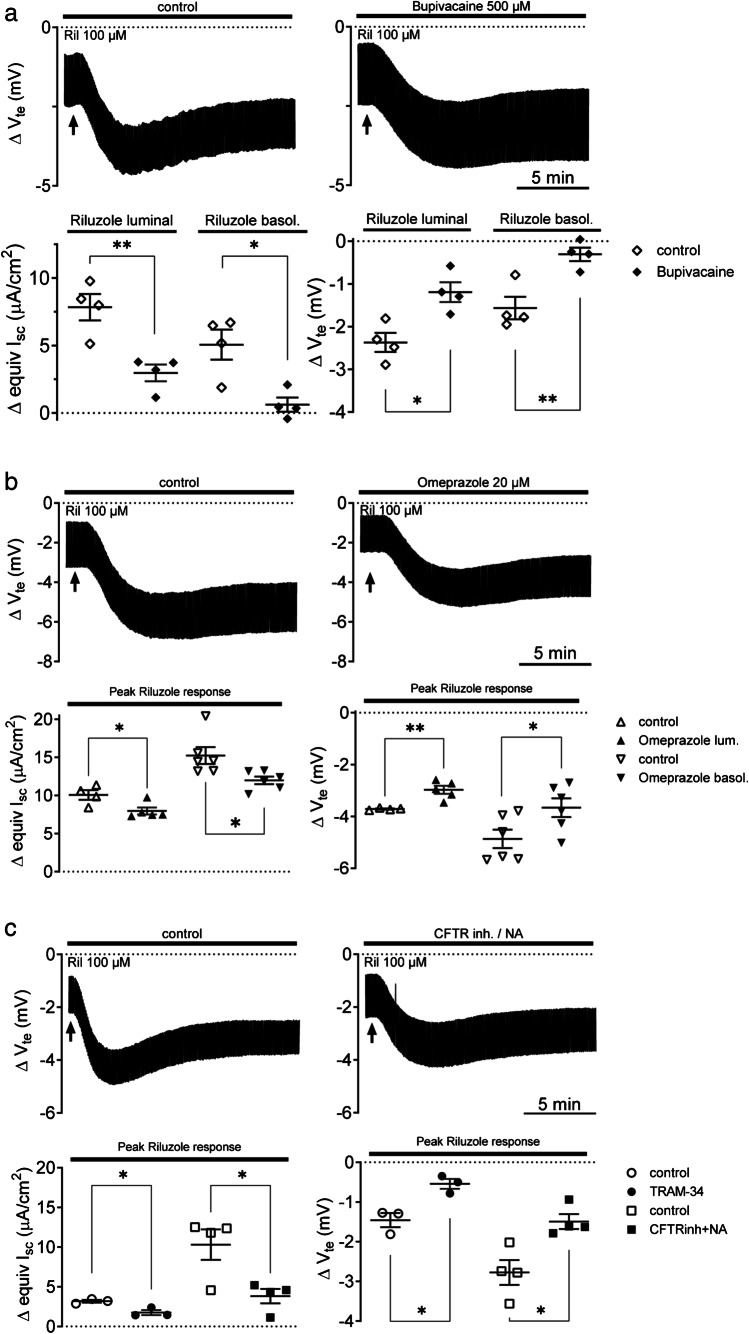
Capan-1 monolayer responses to riluzole stimulation and effects of different inhibitors: **a** riluzole response with and without bupivacaine pretreatment (250 or 500 µM) or **b** pretreatment with omeprazole (20 µM). **c** riluzole response with and without pretreatment with Cl^−^ channel inhibitors (CFTRinh172 and niflumic acid (NA)) and the K_Ca3.1_ channel inhibitor TRAM-34. Number of monolayers tested under the different conditions: *n* = 3–6. *P < 0.05; **P < 0.01

To characterize the contribution of riluzole-induced K^+^ channel activation to epithelial anion secretion, we tested whether riluzole produces electrogenic responses from Capan-1 monolayers in the Ussing chamber setup and whether these could be affected by K2P and KCa3.1 channel as well as chloride channel inhibitors. Figure 5a shows Capan-1 monolayer responses (equivI_sc_ and *V*_te_ changes) to basolateral and luminal administration of riluzole (100 µM), respectively, with or without luminal bupivacaine pretreatment. Riluzole responses were generally characterized by a slow increase of *V*_te_ towards more negative values with a corresponding increase in the equivI_sc_. This was followed by a slow decline but not a complete return to the resting *V*_te_ and equivI_sc_ levels. Scatter plots summarize the mean peak equivI_sc_ and *V*_te_ changes due to either luminal or basolateral addition of riluzole. Luminal bupivacaine pretreatment reduced these peak responses from Capan-1 monolayers significantly, both after riluzole application to the luminal ((7.8 ± 1.0 versus 3.0 ± 0.6 μA/cm^2^ (*n* = 4) (*P* = 0.006) and − 2.4 ± 0.2 versus − 1.2 ± 0.2 mV (*n* = 4) (*P* = 0.011)) or basolateral compartment ((5.1 ± 1.1 versus 0.6 ± 0.5 μA/cm^2^ (*n* = 4) (*P* = 0.011) and − 1.6 ± 0.3 mV versus − 0.3 ± 0.2 mV (*n* = 4) (*P* = 0.006)).

In the following, we tested whether omeprazole (20 µM) influenced riluzole responses from Capan-1 monolayers in order to estimate whether these are linked to H^+^,K^+^-ATPase activity. Figure 5b shows the decrease of riluzole-induced equivI_sc_ and *V*_te_ changes after luminal omeprazole pretreatment ((8.0 ± 0.5 (*n* = 5) versus 10.1 ± 0.6 (*n* = 4) μA/cm^2^ (*P* = 0.027) and − 3.0 ± 0.2 (*n* = 5) versus − 3.7 ± 0.02 mV (*n* = 4) (*P* = 0.004)). Basolateral pretreatment with the proton pump inhibitor also lowered riluzole-induced equivI_sc_ and *V*_te_ changes ((12.0 ± 0.5 versus 15.2 ± 1.1 μA/cm^2^ (*n* = 6) (*P* = 0.022)) and − 3.7 ± 0.4 versus − 4.9 ± 0.4 mV (*n* = 6) (*P* = 0.038)), implying that H^+^,K^+^-ATPase activity is necessary for K^+^ recirculation or that K2P activity is affected by pH changes.

Since riluzole is also a potential activator of KCa3.1 channels, which are expressed in Capan-1; we further tested the effect of TRAM-34, which also reduced the responses ((3.2 ± 0.2 versus 1.8 ± 0.3 μA/cm^2^ (*n* = 3) (*P* = 0.017) and (− 1.5 ± 0.2 versus − 0.5 ± 0.1 mV (*n* = 3) (*P* = 0.014)). Finally, we examined whether the electrogenic responses to riluzole were mediated by an increase in driving force for anion transport from Capan-1 monolayers via Cl^−^ channels. Figure 5c shows that pretreatment of Capan-1 cells both with CFTRinh172 and niflumic acid, inhibitors of CFTR and Ca^2+^ activated Cl^−^ channels, respectively, inhibited riluzole responses significantly ((10.3 ± 1.9 versus 3.8 ± 0.9 μA/cm^2^ (*n* = 4) (*P* = 0.022) and (− 2.8 ± 0.3 versus − 1.5 ± 0.2 mV (*n* = 4) (*P* = 0.013)).

### K^+^channel transcript expression in Capan-1 conventionally cultured cells versus monolayers

**Fig. 6:**
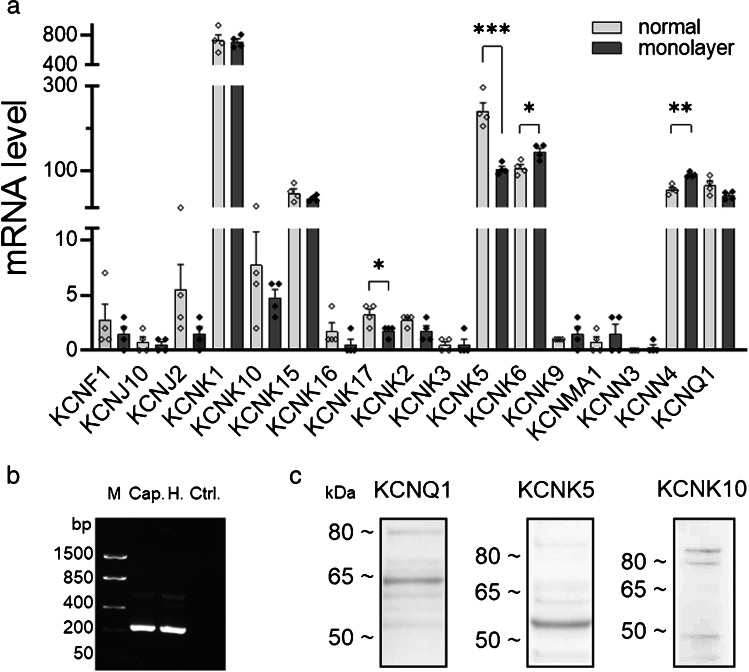
**a** Mean mRNA levels of multiple K channels in conventionally grown Capan-1 cell cultures (light gray bars and symbols) and Capan-1 epithelial monolayers (dark gray bars and black symbols) (*n* = 4 for each condition). Aliases: KCNF1 (Kv5.1), KCNJ10 (Kir4.1), KCNJ2 (Kir2.1), KCNK1 (TWIK-1), KCNK10 (TREK-2), KCNK15 (TASK-5), KCNK16 (TALK-1), KCNK17 (TALK-2), KCNK2 (TREK-1), KCNK3 (TASK-1), KCNK5 (TASK-2), KCNK6 (TWIK-2), KCNK9 (TASK-3), KCNMA1 (KCa1.1), KCNN3 (KCa2.3), KCNN4 (KCa3.1), and KCNQ1 (Kv7.1). **b** RT-PCR analysis of KCNE1 mRNA in cell lysate from Capan-1 (Cap.) (H. refers to HPDE, another cell line). M. refers to the marker and Ctrl. to the control, with template substituted by H_2_O. **c** Western blots of Capan-1 protein samples showing expression of K_v_7.1 (KCNQ1), TASK-2 (KCNK5), and TREK-2 (KCNK10). (Western blot for TREK-1 has been shown previously in Capan-1 cells [54].) *P < 0.05; **P < 0.01; ***P < 0.001

Next, to specify the molecular identity and test if the formation of polarized epithelial monolayers influences their expression, we investigated transcript levels of several K^+^ channels in Capan-1 cells and their variation under different growth conditions. Figure 6a shows a summary of mRNA expression levels of multiple K^+^ channels in Capan-1 cells, either cultured conventionally on 6-well plates or grown as epithelial monolayers. The results show that under both growth conditions, mRNA expression levels of KCNK1 (TWIK-1), KCNK6 (TWIK-2), KCNK5 (TASK-2), and KCNK15 (TASK-5) were highest among the K2P channels investigated, whereas mRNA levels of the calcium-activated KCNN4 (KCa3.1) and voltage-activated KCNQ1 (Kv7.1) channels were the highest among the remaining types of examined K^+^ channels. Among the K2P channel subtypes with low levels of mRNA, KCNK10 (TREK-2) transcripts were more abundant than transcripts for KCNK2 (TREK-1), KCNK16 (TALK-1), KCNK17 (TALK-2), and KCNK9 (TASK-3). KCNK3 (TASK-1) mRNA was barely detectable in Capan-1 cells. Among the remaining K^+^ channel types, mRNA levels of KCNJ2 (Kir2.1) were the highest, whereas KCNN3 (KCa2.3) mRNA was almost absent in Capan-1 cells. Comparisons between the mean transcript levels detected in epithelial monolayers versus conventionally cultured Capan-1 cells showed significant downregulation of KCNK5 (TASK-2) and the minimally expressed KCNK17 (TALK-2). In contrast, KCNK6 (TWIK-2) and KCNN4 (KCa3.1) were upregulated in the epithelial monolayers (Fig. 6a). In addition to these channels, we found evidence for mRNA expression of the auxiliary KCNQ1 subunit KCNE1 in Capan-1 cells (expected size 231 bp) (Fig. 6b). Western blotting confirmed the expression of TASK-2, TREK-2, and KCNQ1 further on the protein level in Capan-1 cells (Fig. 6c (and Online Resources 1)). For KCNQ1, a major and a minor band were detected at 65 kDa and 80 kDa, respectively. Regarding TASK-2, a prominent band was observed at 55 kDa, whereas for TREK-2, one band at 50 kDa could be observed and two minor bands around 80 and 115 kDa, respectively. KCa3.1 channel and TREK-1 protein expression in these cells have been earlier reported by our group [23, 54].

### Immunolocalization of the K2P channels TREK-1, TREK-2, and TASK-2 in Capan-1 monolayers

**Fig. 7:**
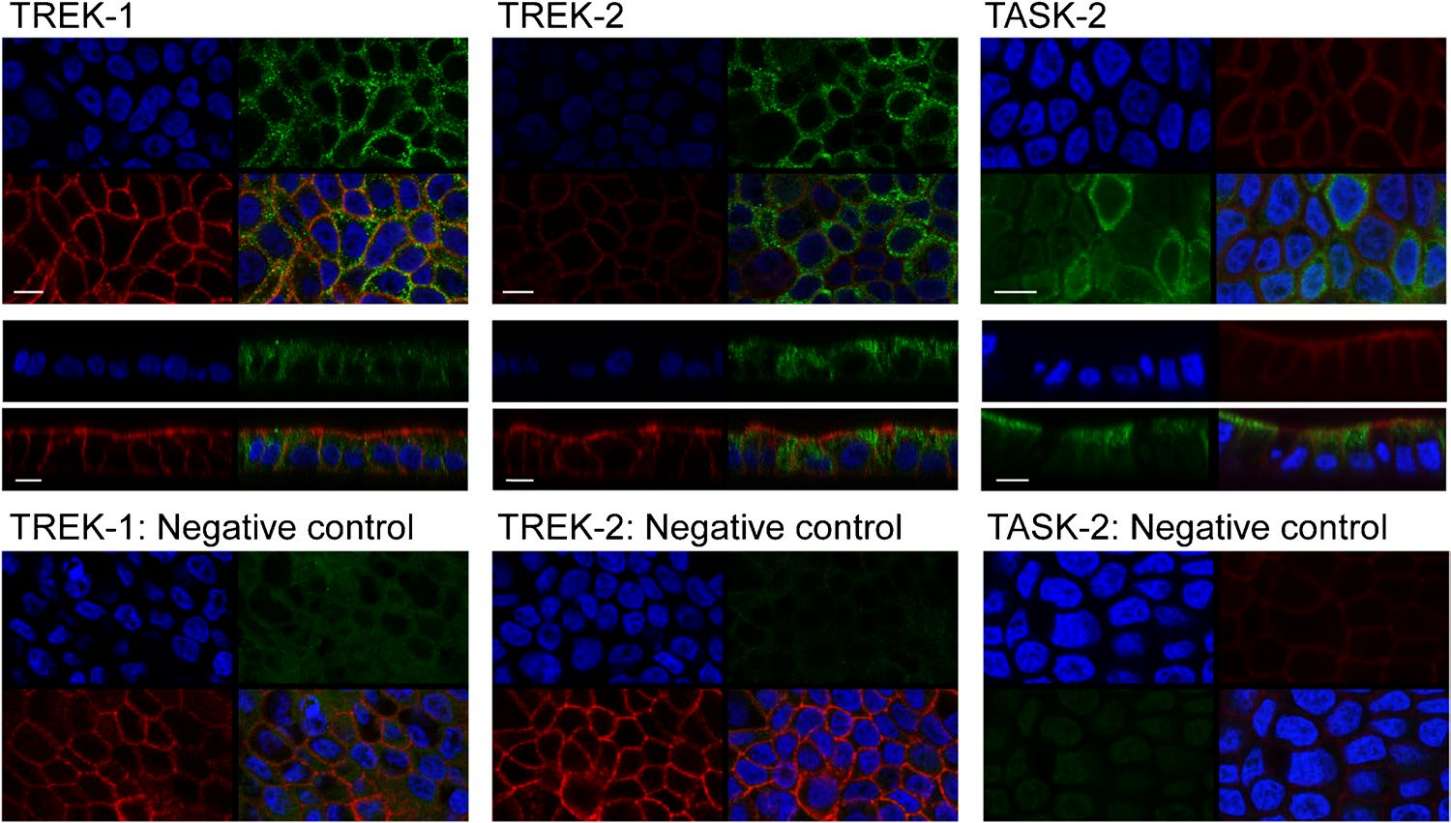
Immunolocalization of TREK-1, TREK-2, and TASK2 K2P channels (green color) in Capan-1 monolayers. Nuclei were stained with DAPI; red color indicates actin staining. Images were obtained in *x*–*y* and *x*–*z* scans. Bar is 10 µm

Since luminal stimulation with riluzole appeared to induce slightly larger anion transport responses and gastric H^+^,K^+^-ATPases are mainly luminally expressed [67], the cellular localization of TASK-2 and both TREK-1 and 2 channels was investigated by immunofluorescence stainings of Capan-1 monolayers (Fig. 7). For the three K2P channel subtypes, staining in proximity to the plasma membrane and intracellular staining was observed. It appeared that fluorescence intensity for TASK-2 was slightly stronger in the luminal cytoplasm and membrane, while it was more laterally located for TREK-1. Interestingly, several of the monolayer embedded cells were devoid of TASK-2 staining, indicating that these cells may not have expressed this K2P subtype on the protein level.

## Discussion

In the present study, we added new characteristics to the Capan-1 duct epithelium model, which are relevant for its employment in studies on human pancreatic epithelial physiology and pathophysiology. Secretory responses were enhanced by agonists stimulating simultaneously the two major intracellular pathways (cAMP and IP_3_/Ca^2+^). We also observed strong responses to histamine as a stimulant that could be inhibited by the H1 antagonist diphenhydramine. Among the physiological agonists tested, the response to carbachol but not histamine could be inhibited by omeprazole. Omeprazole and Cl^−^ channel inhibitors reduced the secretory response to potassium channel openers. Moreover, this study revealed culture-dependent expression of multiple K^+^ channels, including members of the two-pore domain (K2P) potassium channel family.

Our first series of experiments showed that net anion transport (ΔequivI_sc_) was significantly augmented, when Capan-1 monolayers were stimulated with either ATP, carbachol, or histamine, in combination with secretin or VIP. There are several mechanisms by which intracellular pathways can interact. One possibility is the involvement of IRBIT (IP_3_ receptor-binding protein released with IP_3_), a cellular regulator protein that competes with IP_3_ for binding sites on IP_3_ receptors (IP_3_Rs) in the endoplasmatic reticulum [3, 4]. Phosphorylation of IP_3_Rs by cAMP-activated PKA can modulate the receptors in a way that facilitates the release of IRBIT by IP_3_, produced under extracellular (Gq-coupled) receptor stimulation [49]. Unbound IRBIT can then interact with and activate CFTR, the Cl^−^/HCO_3_^−^ exchanger SLC26A6, and the basolateral Na^+^-HCO_3_^−^ cotransporter NBCe1-B, to promote bicarbonate and fluid secretion [49, 58, 62, 74, 75]. Although our observations may agree with IRBIT-mediated synergism between cAMP and IP_3_/Ca^2+^ signaling pathways, a role for IRBIT in ion transport regulation in Capan-1 monolayers has yet to be described. Another way intracellular cAMP and IP_3_/Ca^2+^ signaling could be paired to achieve synergistic responses in Capan-1 monolayers is through direct ion channel stimulation. Many epithelia express cAMP and/or Ca^2+^-stimulated K^+^ channels, e.g., cAMP-activated Kv7.1 (coded by KCNQ1) and calcium-activated KCa3.1 or KCa1.1 channels (coded by KCNN4 and KCNMA1, respectively), in addition to luminal anion channels, including CFTR and/or calcium-activated Cl^−^ channels (CaCC) (e.g., TMEM16/ANO1) (reviewed in [46]). Thus, combined stimulation with agonists that elevate levels of cAMP and Ca^2+^ can activate luminal anion channels, paralleled by basolateral and luminal K^+^ channel activation, thereby increasing the electrical driving force for luminal anion secretion. In Capan-1 monolayers, functional expression of both CFTR and the CaCC TMEM16A/ANO1 [61, 68, 69], as well as KCa3.1 [23], has been reported. Moreover, in the present study, we found evidence for Kv7.1 protein expression in Capan-1 cells and transcript levels that were similarly high as those of KCa3.1 under both growth conditions. Several epithelial cells along the gastrointestinal tract express Kv7.1 and immunolocalization revealed its expression in ductal cells, acini, and islets of Langerhans in the murine pancreas [23]. Nevertheless, Kv7.1 regulation is complex, including sensitivity to membrane voltage and interactions with auxiliary subunits, i.e., members of the KCNE gene family, while the conductance may further vary with the agonists used for stimulation [17]. We did observe KCNE1 (IsK) transcript expression in Capan-1, but further functional studies are needed to reveal the extent to which Kv7.1 and KCNE1 assemble and contribute to anion transport from Capan-1 monolayers. Apart from the above, there is also evidence for functional expression of the calcium sensitive KCa1.1 (BK) channel in native rodent pancreatic ducts [20, 23, 24, 65]. KCa1.1 could be a further candidate for synergistic interactions at the level of intracellular second messengers since the channel is Ca^2+^-activated and its responsiveness to Ca^2+^ can be enhanced by phosphorylation via cAMP-activated PKA [20]. However, in Capan-1, we observed rather low transcript levels of this channel, and it is yet undetermined whether it contributes to anion secretion from these cells. Interestingly, both KCa1.1 and KCa3.1 channels were located in the basolateral and luminal cell membranes of rodent pancreatic ducts [20, 23, 24, 65, 66]. Luminal localization of KCa3.1 was also demonstrated in Capan-1 monolayers [23], which is in line with previous functional descriptions of K^+^ conductance in basolateral and luminal cell membranes of pancreatic ducts [42, 43]. This further agrees with the observation that pancreatic fluid contains potassium, in concentrations that correlate positively with secretory rates [67]. Together, it is likely that at least part of the observed synergism between agonists tested in our study is due to an increased driving force for luminal anion secretion by activation of luminal K^+^ channels expressed in Capan-1, e.g., KCa3.1, and possibly Kv7.1(/KCNE1). It was notable in our experiments that although secretin and VIP concentrations were similar to those tested previously, each alone only produced only weak responses compared to the earlier results from Capan-1 monolayers [69]. Nevertheless, these agonists still induced significant augmentation of responses to luminal ATP, carbachol, and histamine, indicating that secretin and VIP receptors could be activated and that Capan-1 monolayers constitute a useful model for further studies on the synergism between Ca^2+^ and cAMP/PKA signaling pathways. Interestingly, in cystic fibrosis (CF), synergistic airway mucus secretion induced by VIP and carbachol stimulation is lost [7], suggesting this could be of interest for further investigation with the Capan-1 model, concerning CF-associated pancreatic pathology.

Consistent with Capan-1 cell expression of multiple metabotropic P2Y (P2Y1, P2Y2, P2Y4, P2Y6, and P2Y11-14) and ionotropic P2X (P2X1, P2X2, P2X4-X7) purinergic receptors [21], our functional data indicate a significant role for luminal extracellular ATP as a positive modulator of cAMP-mediated hormone or neuropeptide responses of the monolayers. Extrapolated to the organ level, these observations are in line with the suggestion that ATP release from acini and duct cells contributes to the downstream modulation of pancreatic fluid secretion [41]. Since extracellular nucleotides and their hydrolytic breakdown products exert positive and negative effects on target cells and surrounding tissues, purinergic signaling must be tightly controlled [48]. Accordingly, pancreatic duct cells, including Capan-1, express distinct ecto-enzymes, which regulate adequate levels of extracellular nucleotides and nucleosides [30]. Moreover, Capan-1 cells express functional adenosine receptors involved in anion transport [22] and release ATP in response to pH changes and certain disturbances, e.g. osmotic and mechanical stress [30]. Thus, these cells reproduce key aspects of purinergic signaling and synergistic agonist activation, which renders them a valuable model for studies on interactions between extracellular nucleotides and other auto- and paracrine modulators or classical secretagogues.

Among the agonists tested in our study, carbachol and histamine are known stimulants of acid secretion from gastric parietal cells through gastric H^+^,K^+^-ATPases [56]. Since our recent research indicates that expression and function of H^+^,K^+^-ATPases have an influence on pancreatic duct cells under normal and pathophysiological conditions [64, 67], we expected that treatment of Capan-1 monolayers with the proton pump inhibitor omeprazole would affect responses to the agonists above. Nevertheless, omeprazole only induced a slight reduction of carbachol-stimulated ΔequivI_sc_ responses, while the effects of histamine on Capan-1 monolayers were not significantly reduced by the inhibitor. Possibly, relatively short-term omeprazole treatment in these experiments was not sufficient to induce more pronounced inhibition, or its effects may have been spatially confined. Moreover, with the Ussing chamber technique, we cannot evaluate the direct effect of omeprazole on H^+^,K^+^-ATPase activity in Capan-1 due to the electroneutrality of the pump. Nevertheless, the pump inhibition may via changes in the local pH or transmembrane K^+^ gradients affect the secretory responses (see below).

Interestingly, Capan-1 monolayers responded to basolateral histamine stimulation with a relatively large increase in *V*_te_ and corresponding equivI_sc_, often resembling a shape, which agrees with previous responses from monolayers of a dog pancreatic duct cell line [38]. Further experiments have revealed a sensitivity of the histamine-induced *V*_te_ and calcium changes to the H1 receptor antagonist diphenhydramine. Our findings thus indicate that at least one type of histamine receptor is functionally expressed in Capan-1 cells and regulates the secretory response. Histamine, which can be found within the exocrine portion of pancreatic tissue, around blood vessels, and released into pancreatic secretion, has a secretagogue function and contributes likely to the control of pancreatic exocrine secretion, perhaps depending on the species [59]. However, histamine levels and expression may also be increased in inflammation and cancer. For example, depending on the receptor subtypes activated, histamine binding may either inhibit or contribute to pancreatic cancer cell proliferation, as indicated by studies on the human PDAC cell line PANC-1 [8, 9]. Moreover, in the inflamed pancreas, the distribution and content of histamine seem to differ compared to normal conditions [59]. Thus, histamine signaling, metabolism, and interactions with other hormones and neurotransmitters may represent new targets for research into the pathogenesis of pancreatitis and pancreatic ductal adenocarcinoma [16, 59].

A further aim of this study was to characterize K^+^ channel gene expression and function in Capan-1 monolayers with a particular interest in candidates, which we hypothesized could cooperate with H^+^,K^+^-ATPases in pH balancing and K^+^ homeostasis and support anion secretion. Apart from the above-discussed KCa3.1 and KCNQ1 channels with relevance for stimulated bicarbonate transport, we detected gene transcripts for several members of the K2P potassium channel family that could be involved in pH sensing, K^+^ recycling, and maintenance of resting cell membrane potential (*V*_m_). Thus, among alkaline activated K2P channels, Capan-1 cells express particularly high levels of mRNA for TASK-2 (KCNK5, K_2P_5.1), a member of the TALK K2P subfamily that is activated by external alkalinization [40] and shows a broad peripheral expression that includes the human pancreas and other bicarbonate transporting epithelia [35, 52]. In the kidney, for example, results from a study on knockout mice suggest that alkaline activation of TASK-2 supports proximal tubular bicarbonate reabsorption [70]. Thus, it is conceivable that TASK-2 activity in the exocrine pancreas could be related to bicarbonate transport. With 50% of the maximal current occurring at pH 8 [40], the channel is presumably only weakly open under resting pH conditions. Upon contact with the forming alkaline secretion, the pH of which can exceed 8 [46], TASK-2 opening would enable *V*_m_ hyperpolarization that sustains further efflux of bicarbonate from ductal cells, e.g., when the driving force declines. It is possible then that the released K^+^ is reabsorbed via luminal H^+^,K^+^-ATPases, preventing the buildup of K^+^ that could compromise the transepithelial potential. The protons released in exchange for K^+^ may counteract excessive alkalinity and potential caustic effects of the bicarbonate-rich fluid on the cells, as previously suggested [45]. However, in the present study, we were not able to demonstrate that alkalinization of the luminal solution alone significantly enhanced responses from Capan-1 monolayers to stimulation. Hyperpolarization likely occurs fast in response to alkalinization, and our present approach was not sufficient to reveal any immediate effect on anion transport from Capan-1. Further studies are also needed to confirm the above suggestion that TASK-2 cooperates with H^+^,K^+^-ATPases, as the present Capan-1 monolayer model is limited. Interestingly, TASK-2 channel expression has also been described in the HPAF human PDAC cell line [15], but not much is known about the differential regulation of the channel in normal versus diseased pancreatic tissue. Results from our transcript analyses suggest that growth conditions significantly alter KCNK5 transcript expression. One explanation could be that TASK-2 expression is downregulated in response to the accumulation of metabolites, such as lactic acid, which may occur with increasing tight junction formation and transepithelial resistance upon the formation of monolayers.

Apart from TASK-2, we also observed protein expression of TREK-1 and TREK-2, which are members of the mechanosensitive TREK subfamily of K2P channels. However, their transcript levels in Capan-1 were low in the present study, which agrees with previously shown low TREK-1 mRNA abundance in human pancreatic tissue but contrasts with some higher levels shown for TREK-2 [31, 35]. Since mRNA abundance does not always correlate well with protein levels [33], we still assume the expression of both TREK channels in Capan-1. Although the two K2P subunits display about 63% sequence identity [31], their activities are inversely affected by extracellular pH changes; i.e., TREK-1 is inhibited by acidic extracellular pH, while TREK-2 is activated [53]. Moreover, the two subunits can heterodimerize with each other and form channels that differ from homodimers in voltage and external pH sensitivity, showing activation by acidic and alkaline conditions and low activity in the physiological range of extracellular pH [32]. Since TREK-1 and TREK-2 are opened by intracellular protons [5, 27, 31, 34], they could also be considered as potential partner K^+^ channels for H^+^,K^+^-ATPases, releasing K^+^ ions in response to intracellular acidification, which can be reabsorbed through the pumps in exchange with intracellular protons. Interestingly, TREK channels also open in response to osmotic and mechanical stimuli, for example, membrane stretch, [5, 31, 34, 50]. These channels could thereby sense osmotic stress and pressure due to intraluminal fluid volume increase as well as shear forces that occur during the flow of the pancreatic secretion through the ductal system. Thus, we propose that their expression in pancreatic duct cells may be relevant for K^+^ recirculation, pH adjustment, and background conductance under resting conditions and in response to physicochemical stimuli that can impose a severe challenge on ductal cells. We further aimed to establish a functional role of these channels in anion transport from Capan-1 monolayers. However, functional characterization of endogenous K2P channels is complicated by the lack of specific agonists and inhibitors and further compounded by overlapping subtype expression and the tendency to form heterodimers with alternative regulation and pH sensitivities [32], as discussed above. Nonetheless, the local anesthetics, bupivacaine, and lidocaine have been shown to inhibit TASK-2, TREK-1, and TREK-2 channels [26, 28, 29, 37, 51, 57]. Based on the literature, we took inspiration from a study on monolayers of Calu-3 cells, in which these agents reduced basal anion secretion [10]. In Capan-1 monolayers, bupivacaine and lidocaine pretreatment had a significant impact on agonist-induced responses and basal anion secretion (lidocaine), indicating that one or more of these K2P channel subtypes may be functional and contribute to K^+^ conductance in Capan-1 cells, inhibition of which reduces the driving force for luminal anion exit. Following the study on Calu-3 cells [10], we also tested the neuroprotectant riluzole with and without bupivacaine pretreatment on Capan-1 cells. Riluzole transiently activates both TREK-1 and TREK-2 [13, 31], and consistent with K^+^ channel opening, our voltage dye experiments showed that the agent induced hyperpolarization of the cell membrane potential, which was reduced after bupivacaine pretreatment. In Capan-1 monolayers, riluzole alone increased the resting luminal anion flux, and this response was also sensitive to bupivacaine, suggesting the participation of either TREK-1, TREK-2, or both in the response. Pretreatment with the proton pump inhibitor omeprazole also led to some reduction in the electrogenic secretory responses to riluzole. One feasible explanation is that due to inhibited reabsorption by H^+^,K^***+***^-ATPases, a local buildup of K^+^ ions near the luminal membrane decreased the outward gradient for K^+^ secretion which, in turn, could lower driving force for anion efflux under the influence of riluzole. Our findings indicate that in the absence of hormonal, neurotransmitter, or local paracrine stimulation, TREK-1 and/or TREK-2 channels, and possibly further riluzole-responsive K^+^ channels, could be involved in K^+^ recycling coupled to proton export via H^+^,K^+^-ATPases, to prevent extensive loss of K^+^ through background K2P channels and associated depolarization due to intraluminal K^+^ accumulation, as discussed above. Since none of the inhibitors completely eliminated the response, we considered further targets of riluzole. KCa3.1 channels are also known to be activated by the agent [72]. Accordingly, treatment of Capan-1 monolayers with the Kca3.1 channel inhibitor TRAM-34 [73] significantly decreased anion transport after the application of riluzole. However, the opening of KCa3.1 channels by riluzole would require the presence of certain amounts of intracellular calcium [72], and therefore, it seems unlikely that this is the only K^+^ channel type involved in the monolayer response, in the absence of Ca^2+^ mobilizing agonists. Therefore, we suggest that anion transport from Capan-1 monolayers following riluzole application is a compound response that includes activation of K2P channel subtypes TREK-1 and/or TREK-2 and KCa3.1 channels, which increase the driving force for anion exit through luminal Cl^−^ channels. Involvement of the latter was critical as demonstrated by the reduced responses after treatment with Cl^−^ channel inhibitors and may be related to a rise in intracellular cAMP riluzole can induce [13]. Interestingly, a recent study has investigated the potential influence of riluzole on pancreatic cancer cells, showing that treatment of PDAC cell lines with the agent inhibited migration and proliferation, promoting apoptosis and inhibiting autophagy [63]. Apart from such long-term effects, the results from our study suggest that riluzole has also immediate effects on PDAC cell behavior through its influence on ion transport, in particular K^+^ conductance, and thereby cell membrane potential. Thus, it could be of interest to use the current Capan-1 monolayer model to further explore the potential short- and long-term impact of riluzole on human pancreatic adenocarcinoma cells.

Lastly, the present study showed that Capan-1 cells expressed high levels of transcripts for the K2P channels TWIK-1 (KCNK1), TWIK-2 (KCNK6), and TASK-5 (KCNK15), in contrast to TALK-1 (KCNK16), and TALK-2 (KCNK17), as well as TASK-1 (KCNK3), the mRNA levels of which were low. Our observations differ from previously reported low levels of TWIK-1 and high levels of TASK-1 mRNA in the human pancreas [35]. This inverse expression pattern could be related to inherent characteristics of Capan-1 cells, originating from pancreatic cancer [11], as they agree with high TWIK-1 and low TASK-1 transcript levels described from pancreatic adenocarcinoma [25, 71]. Moreover, the low TALK-1 and 2 transcript levels in Capan-1 are in contrast to previously reported abundant expression of both K2P subunits in the human pancreas [18]. These members of the TALK K2P channel subfamily are, similar to TASK-2, sensitive to pH changes and activated at alkaline pH [18]. Thus, additional studies are needed to understand whether their low transcript expression could be a further characteristic of Capan-1 cells. On the other hand, our observation of abundant TWIK-2 (KCNK6) mRNA expression in Capan-1, which was even higher in monolayers, is in line with the previously reported high transcript levels in the human pancreas [35]. Lastly, TASK-5 transcript levels were also relatively high in Capan-1; however, it is not clear to which extent this subunit can produce K^+^ channel activity, and it is regarded as silent, which might be due to the requirement of yet unidentified cofactors that would promote its functional expression [14]. Together, further research is necessary to understand the roles of the above-identified K2P channels with their unique pH sensitivities and regulation in pancreatic duct cell physiology.

## Conclusions

From the perspective of modeling mechanisms in pancreatic bicarbonate transport, Capan-1 cells, grown as polarized monolayers, maintain considerable functional resemblance to normal human pancreatic duct epithelium. Based on our present observations, we conclude that they constitute a relevant model for further studies on synergistic interactions between physiological agonists. Furthermore, our study indicates functional coupling between K2P channels and acid–base transport and secretory responses of the ductal epithelium. From the perspective of their origin, Capan-1 cells and monolayers display some divergence in transcript profiles with respect to potassium channel subtypes. Thus, the high level of KCa3.1 channel mRNA and the inverse TWIK-1 and TASK-3 transcript expression compared to normal pancreatic tissue may be related to their evolution as pancreatic adenocarcinoma cells, and therefore, Capan-1 grown as epithelium may also have limitations concerning pancreatic duct physiology.

## Supplementary Information

Below is the link to the electronic supplementary material.

Supplementary file1 (PDF 427 KB)

## Data Availability

The datasets that support the findings of this study are available from the corresponding author upon reasonable request.
