# Supplementary material for: Synergistic effects of agonists and two-pore-domain potassium channels on secretory responses of human pancreatic duct cells Capan-1
Source: Pflugers Arch. 2022 Dec 19;475(3):361–79. doi: 10.1007/s00424-022-02782-9 (PMC9908661; doi:10.1007/s00424-022-02782-9)

Synergistic effects of agonists and two-pore-domain potassium channels on secretory responses of human pancreatic duct cells Capan-1.

Sørensen, C.E.*[1,2], Trauzold, A., Christensen, N.M., Tawfik, D., Szczepanowski, M., Novak, I.
*[1]Section for Cell Biology and Physiology, Department of Biology, [2]Section for Clinical Oral Microbiology, Department of Odontology, Faculty of Health and Medical Sciences, University of Copenhagen, Copenhagen, Denmark (*corresponding author)

Online Resource 1:
Original Western blots for TASK-2, KCNQ1 and TREK-2 in two different lysate samples from Capan-1 cells.
Results shown for HPDE and Panc-1 samples are unrelated to this study.

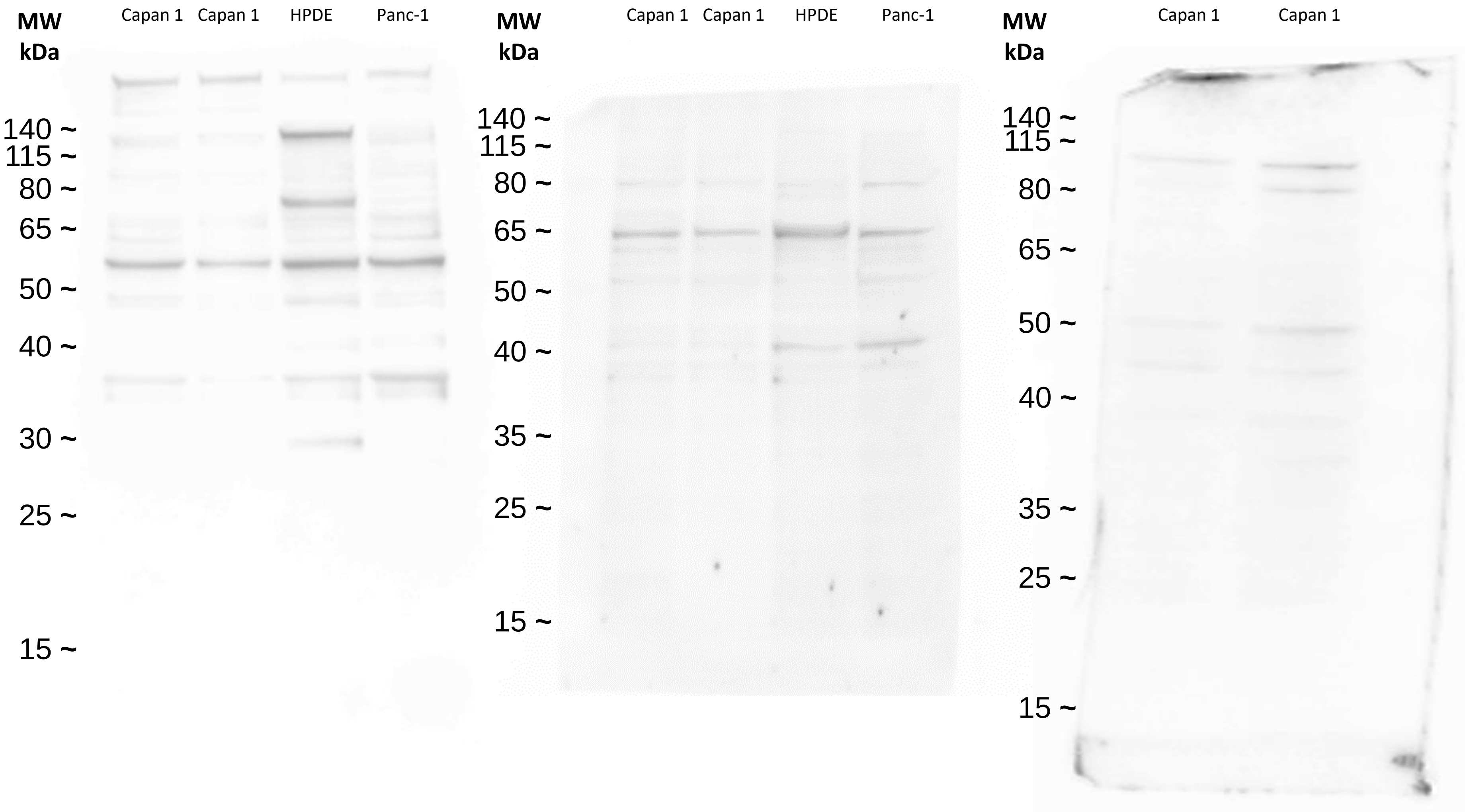

Supplement: Supplementary file 1 — Supplementary file1 (PDF 427 KB) [file 424_2022_2782_MOESM1_ESM.pdf]
